# LncRNA MALAT1 exhibits positive effects on nucleus pulposus cell biology in vivo and in vitro by sponging miR-503

**DOI:** 10.1186/s12860-020-00265-2

**Published:** 2020-03-30

**Authors:** Hongyu Zheng, Tingting Wang, Xiangmin Li, Wei He, Zhiqiang Gong, Zhenkai Lou, Bing Wang, Xingguo Li

**Affiliations:** 1 Department of Emergency Medical, First Affiliated Hospital of Kunming Medical University, Kunming, China; 2 Department of Geriatrics, Yan’ An Hospital of Kunming City, Kunming, China; 3 Department of Panicaceae, First Affiliated Hospital of Kunming Medical University, Kunming, China; 4 Department of Orthopedics, Qianjiang Central Hospital, Qianjiang, China; 5 Department of Orthopedics, First Affiliated Hospital of Kunming Medical University, No. 295 Xichang Road, Kunming, Yunnan China

**Keywords:** Intervertebral disc degeneration, Long noncoding RNA MALAT1, microRNA-503, Nucleus pulposus cells, Apoptosis, MAPK pathway

## Abstract

**Background:**

Intervertebral disc degeneration (IDD) is characterized by the loss of nucleus pulposus cells (NPCs) and phenotypic abnormalities. Accumulating evidence suggests that long noncoding RNAs (lncRNAs) are involved in the pathogenesis of IDD. In this study, we aimed to investigate the functional effects of lncRNA MALAT1 on NPCs in IDD and the possible mechanism governing these effects.

**Results:**

We validated the decreased expression of MALAT1 in the IDD tissues, which was associated with decreased Collagen II and Aggrecan expression. In vitro*,* overexpressed MALAT1 could attenuate the effect of IL-1β on NPC proliferation, apoptosis, and Aggrecan degradation. In vivo*,* MALAT1 overexpression attenuated the severity of disc degeneration in IDD model rats. Our molecular study further demonstrated that MALAT1 could sponge miR-503, modulate the expression of miR-503, and activate downstream MAPK signaling pathways. The effects of MALAT1 on NPCs were partially reversed/aggregated by miR-503 mimics/inhibitor treatment.

**Conclusion:**

Our data suggested that the MALAT1-miR-503-MAPK pathway plays a critical role in NPCs, which may be a potential strategy for alleviating IDD.

## Background

Intervertebral disc degeneration (IDD) has been widely regarded as making a significant contribution to low back pain (LBP), a leading cause of chronic pain, at various times and is an important cause of a series of spinal degenerative diseases [1]. The intervertebral disc (IVD), consists of three structurally connected parts: the peripheral annulus fibrosus (AF), the central gelatinous nucleus pulposus (NP) and the cartilage endplates (CEPs) [2], and it is the largest avascular organ. Nucleus pulposus cells (NPCs) are highly hydrated in healthy IVDs, which can produce abundant Aggrecan and Collagen II [3] and can ensure the IVD mechanical function of distributing the axial compressive forces acting on the spine and the multiaxial flexibility together with AF, cartilaginous and bony endplates [4]. Loss of NPCs [5, 6] and imbalance of matrix synthesis and degradation [7], play important roles in the occurrence and development of IDD. Therefore, targeting the function of NPCs represents a potential strategy for the improvement of IDD.

Long noncoding RNAs (lncRNAs) are a class of noncoding RNAs with a transcriptional length of more than 200 nucleotides, regulating gene expression in epigenetics, transcription, and post-transcription [8]. Recently, accumulating evidence has shown that aberrantly expressed lncRNAs play a vital role in the IDD process. The levels of MALAT1 were significantly reduced in NPCs from IDD patients [9]. Recent reports found that MALAT1, metastasis-associated lung adenocarcinoma transcript-1, promoted caspase 3 activity, regulated the secretion of cytokines, and was involved in cell proliferation, migration, and apoptosis [10, 11]. These findings suggested that MALAT1 might participate in IDD development by inducing NPCapoptosis and the secretion of pro-inflammatory cytokines. However, little is known about the role and mechanism of MALAT1 in IDD.

The genetic mechanisms of lncRNAs primarily include miRNAs sponges. LncRNAs could posttranscriptionally interact with miRNAs to serve as competing endogenous RNAs (ceRNAs), thereby repressing miRNA expression, and can inhibit translation or degradation of miRNA downstream targets. Yan et al. [12] demonstrated that MALAT1 could directly bind to miR-503 and modulate the expression of miR-503. miR-503, located on the chromosome Xq26.3, is an intragenic miRNA and belongs to the miR-16 family [12]. Prevailing evidence suggests that miR-503 exerts diverse biological functions, such as osteoblast proliferation and apoptosis, which are potentially amenable to therapeutic manipulation for clinical application [13]. Additionally, in several cell lines, MALAT1 could regulate downstream MAPK and activator protein-1 (AP1) signaling pathways, which play a critical role in intervertebral disc degeneration [14–16]. However, the impact of MALAT1 on the MAPK/AP1 pathway in NPC has not been determined.

In the present study, we found lower levels of MALAT1 expression in IDD tissues and an association with Collagen II/Aggrecan. We also analyzed the functional effects of MALAT1 overexpression, and miR-503 mimics/inhibitor on NPC proliferation, apoptosis, and ECM degradation in vitro and in vivo. In addition, we investigated the involvement of the MAPK/AP1 signaling pathway in this process.

## Results

### Expression of MALAT1 in lumbar IDD tissues and the correlation with the prognosis of IDD

**Fig. 1:**
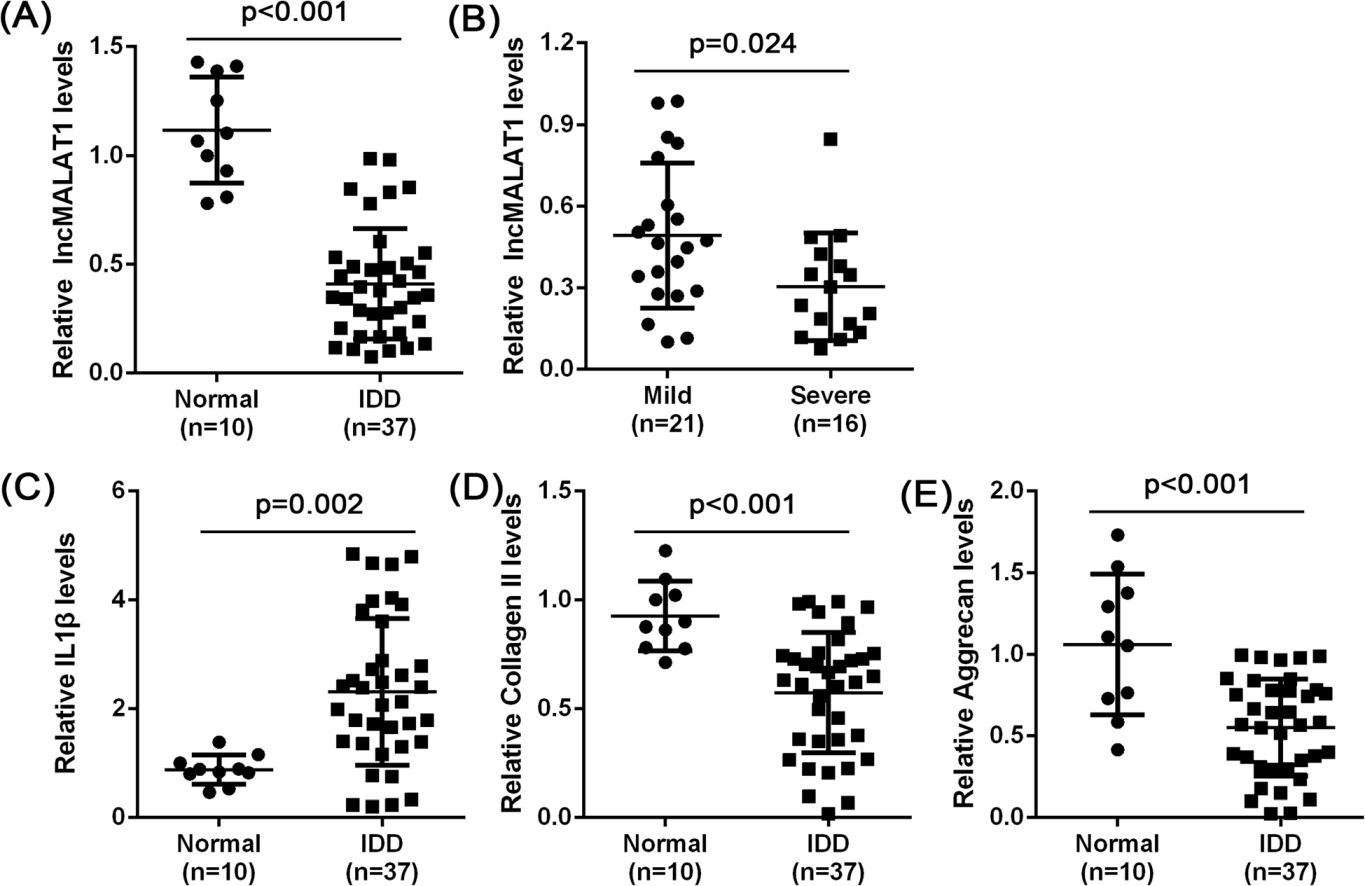
The expression of MALAT1 in lumbar IDD tissues and the correlation with prognosis of IDD **a** MALAT1 expression in 10 normal and 37 IDD tissue specimens determined by real-time PCR assays. **b** MALAT1 expression in 37 IDD tissue specimens was delineated according to the Pfirrmann grading groups (mild *n* = 21, severe *n* = 16). **c**–**e** The mRNA expression of IL-1β, Collagen II and Aggrecan in 10 normal and 37 IDD tissue specimens was determined using real-time PCR assays. The data are presented as mean ± SD of three independent experiments

To investigate the effect of lnc-MALAT1 in IDD, we examined its expression in tissue specimens, including 10 normal specimens and 37 lumbar IDD specimens. The results showed that MALAT1 expression was significantly down-regulated in the lumbar IDD specimens (Fig. 1a). We evaluated the progression of the above 37 IDDs according to the Pfirrmann grading (mild degeneration: 3–4, 21, severely degraded: 5–6, 16). The MALAT1 level was lower in severely IDD specimens than in mild IDD specimens (Fig. 1b). IDD is characterized by decreased cell number, increased matrix degradation [17] and increased proinflammatory cytokine release [18]. Therefore, we examined the expression of IL-1β, Collagen II and Aggrecan in IDD specimens and normal specimens. Compared with normal specimens, IL-1β mRNA levels were significantly up-regulated in IDD specimens, while Collagen II and Aggrecan levels were significantly down-regulated (Fig. 1c-e). This result suggests that MALAT1 may play a potential role in IDD progression.

### IL-1β inhibits the function of NPCs and the expression of MALAT1

**Fig. 2:**
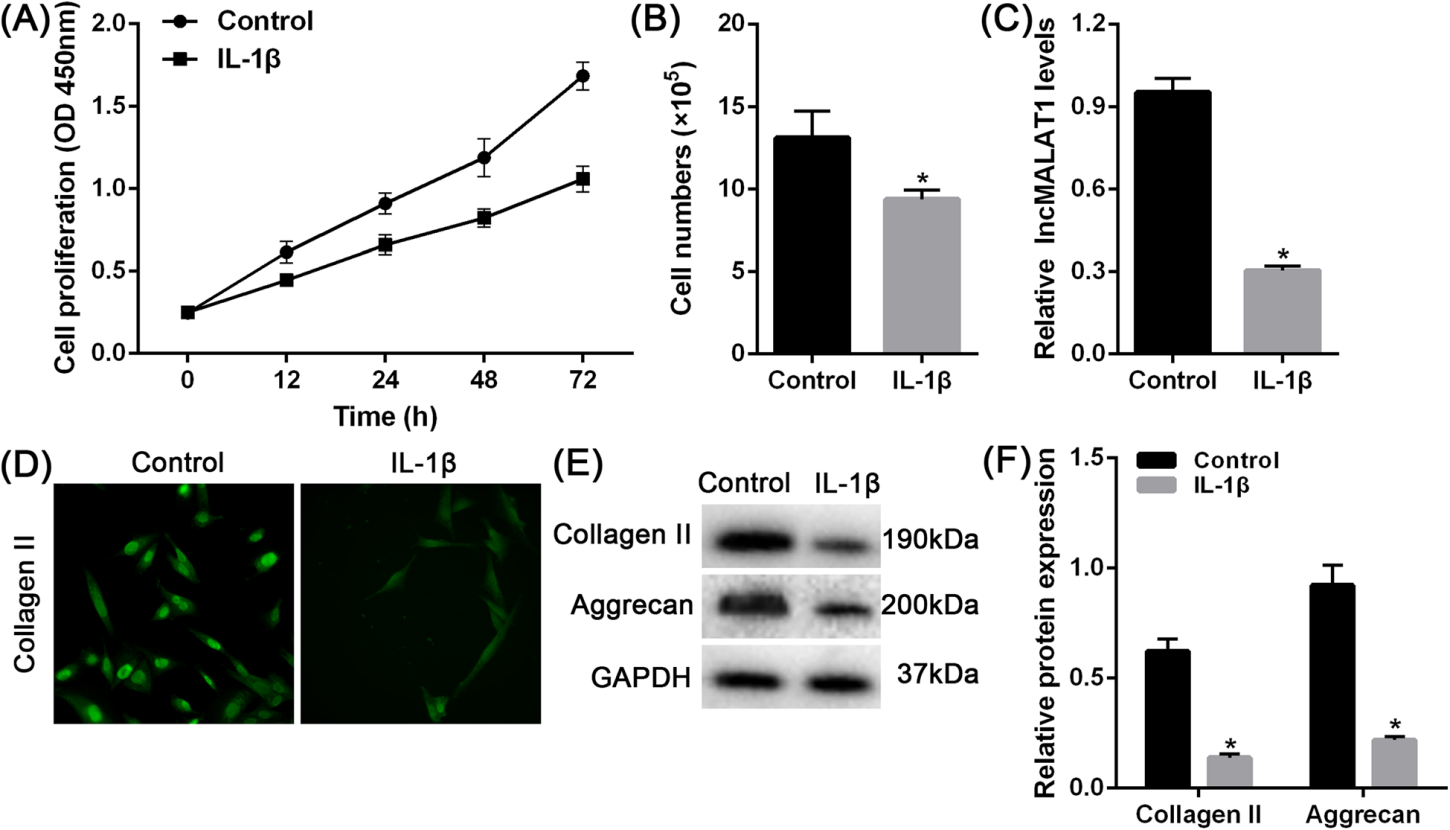
The expression and function of MALAT1 in IL-1β-stimulated NPC. **a** cell proliferation and **b** cell numbers of human NPCs in response to IL-1β stimulation were detected (scale bars, 50 μm). **c** The expression of MALAT1 in response to IL-1β stimulation was determined using real-time PCR assays. **d** The content of Collagen II in human NPCs (NPCs) in response to IL-1β stimulation was determined using Immunofluorescence staining assays. **e**-**f** The protein levels of Collagen II and Aggrecan in NPCs in response to IL-1β stimulation were determined using Immunoblotting assays. The data are presented as mean ± SD of three independent experiments. *P < 0.05, compared to control group

Elevated levels of proinflammatory mediators increased Aggrecan and Collagen II degradation, and increased degradation of extracellular matrix (ECM) has been widely regarded as a significant contributor to intervertebral disc degeneration (IDD). After stimulation with IL-1β, the proliferation and number of human NPCs were inhibited (Fig. 2a-b). Additionally, IL-1β reduced the expression of MALAT1 (Fig. 2c). We found that IL-1β can reduce the expression of Aggrecan and Collagen II (Fig. 2d-f).

### MALAT1 modulates IL-1β-induced dysfunction of NPC proliferation, apoptosis, and ECM degradation in vitro

**Fig. 3:**
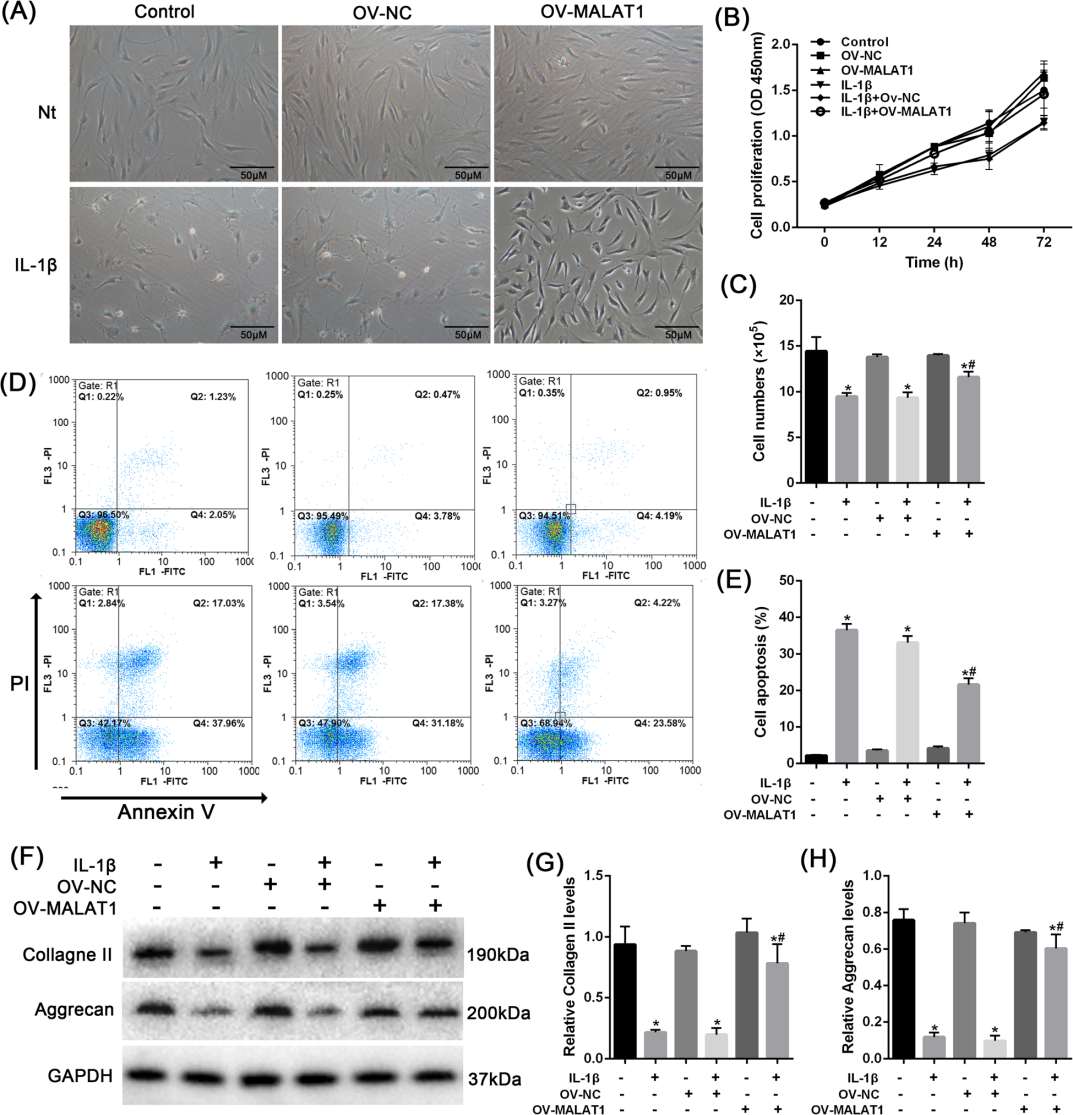
The effect of MALAT1 on NPCs proliferation, apoptosis, and ECM degradation **a** NPCs were transfected with OV-NC or OV-MALAT1 under IL-1β stimulation, the cell was observed. **b** and **c** The cell viability was determined using CCK8 assays and cell counting. **d** and **e** The cell apoptosis was determined using Annexin V/PI staining. **f**-**h** The protein levels of Collagen II and Aggrecan were determined using Immunoblotting assays. The data are presented as mean ± SD of three independent experiments *P < 0.05, compared to control group; #P < 0.05, compared to IL-1β + OV-NC (negative control) group

Next, the function of MALAT1 in IL-1β-stimulated NPCs was evaluated by measuring Collagen II and Aggrecan protein levels, cell proliferation, and cell senescence in human NPCs (Fig. 3a). MALAT1 overexpression reversed the effect of IL-1β on NPC proliferation (Fig. 3b), NPC number (Fig. 3c), and cell apoptosis (Fig. 3d-e) induced by IL-1β. Overexpression of MALAT1 reversed the inhibitory effect IL-1β on Collagen II and Aggrecan protein levels (Fig. 3f-h). These data indicate that MALAT1 overexpression partially reverses the effect of IL-1β on NPC.

### MALAT1 modulates IL-1β-induced IDD and inhibits apoptosis in the rat model

**Fig. 4:**
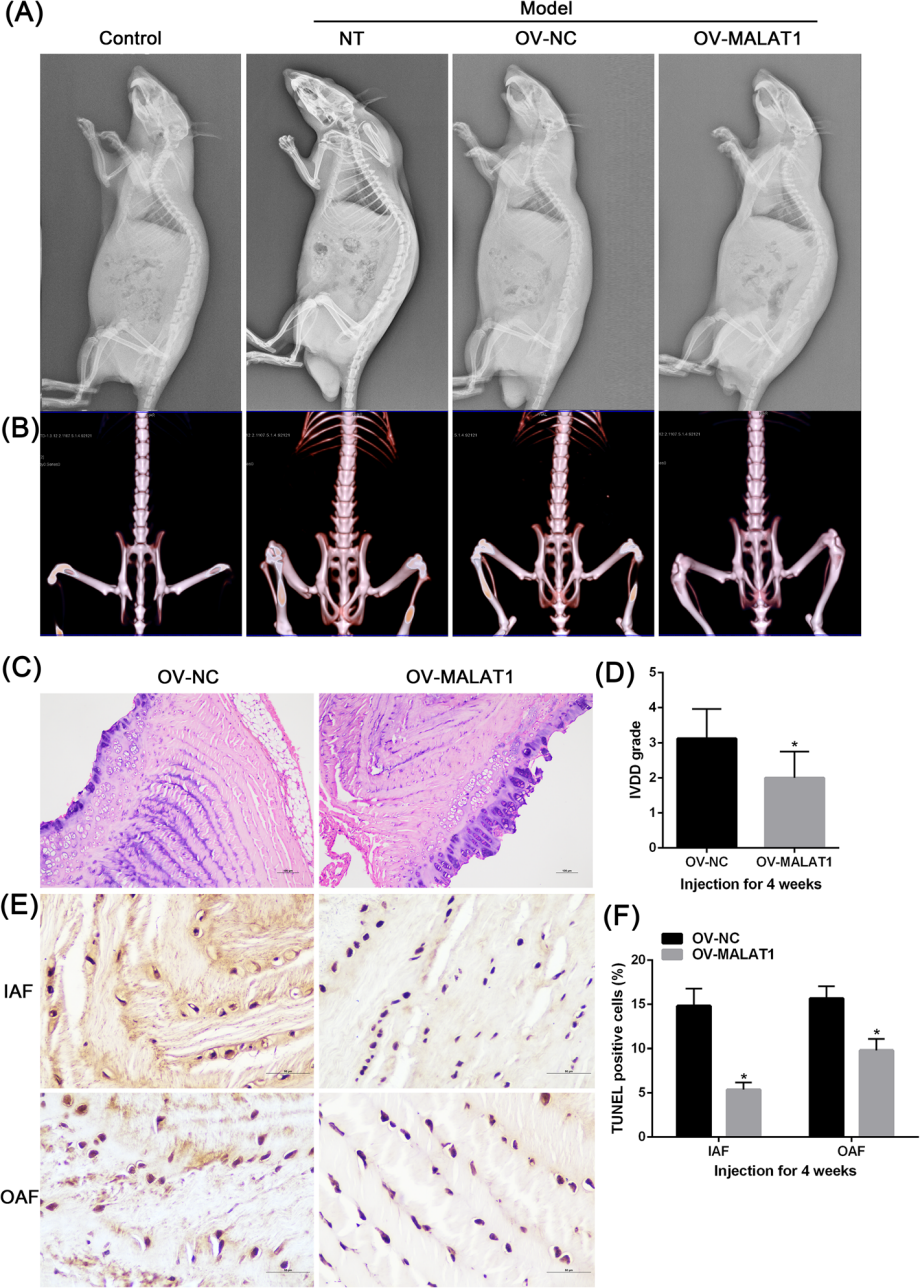
The function of MALAT1 in vivo (**a**) Lateral radiographies of the rat lumbar spine and disc height index (DHI) in control groups andMALAT1 group. **b** MRI at seven weeks after injection and change of MRI grade in control groups and MALAT1 group. **c**-**d** HE staining and comparison of the grade of IDD control groups and MALAT1 group. **e**-**f** Cell apoptosis according to in situ TUNEL staining. The data are presented as mean ± SD of three independent experiments. *P < 0.05, compared to OV-NC group

After 4 weeks of modeling, characterization of rat lumbar vertebral IDD caused by unbalanced dynamics and static forces was observed on X-ray films. After the injection of MALAT1 into the L4/L5 and L5/L6 discs of the rats, there was no significant difference between the two groups (Fig. 4a). At 4 weeks after injection, the MRI of the intervertebral disc in the OV-MALAT1 group showed stronger signal intensity than the control group (Fig. 4b).

The intervertebral disc AF of the OV-MALAT1 group showed normal structure or mild serpentine appearance, while the control group showed mild to moderate appearance, eventually showing a serious serpentine appearance, contour reversal, and rupture (Fig. 4c). At four weeks after injection, the histological score of the Ov-MALAT1 group was significantly lower than that of the OV-NC group (*P* < 0.05) (Fig. 4d). The apoptosis of intervertebral disc cells was detected by in situ TUNEL staining. After four weeks of injection, the ratio of apoptotic cells in the annulus fibrosus (external or internal) of the OV-MALAT1 group was significantly lower than that of the OV-NC group (Fig. 4e-f).

### MALAT1 acts as a sponge for miR-503

**Fig. 5:**
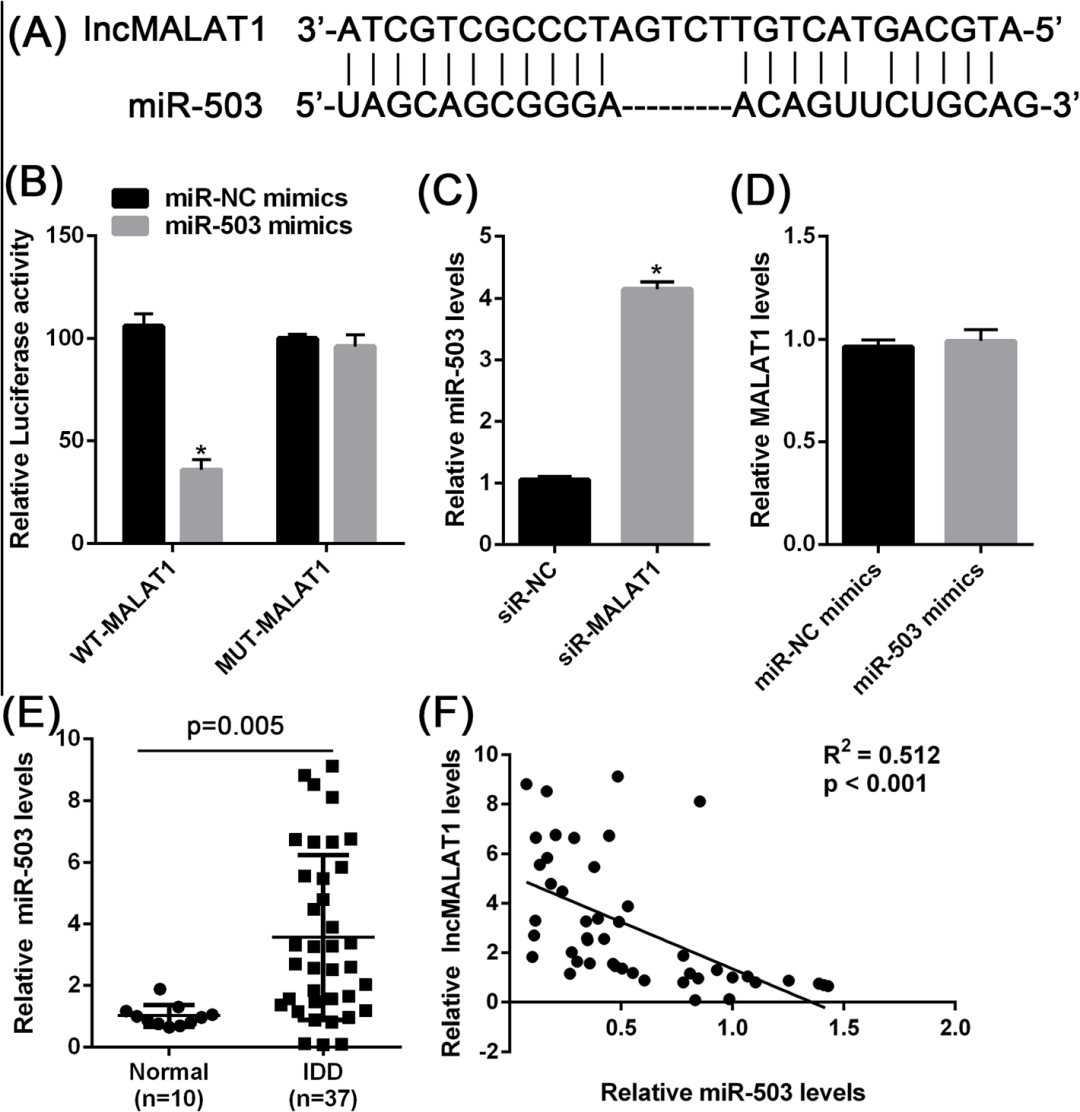
MALAT1 acts as a sponge for miR-503 (**a**) Schematic representation of binding sites between MALAT1 and miR-503 predicted by StarBase software. **b** Wild-type and mutant-type MALAT1 luciferase reporter gene vectors were constructed and named wt-MALAT1 and mut-MALAT1; mut-MALAT1 contained a 4 bp mutation on any of the predicted miR-503 binding sites. The above vectors were co-transfected into HEK-293 cells with miR-NC or miR-503 mimics; the luciferase activity was determined. **c** NPCs were transfected with MALAT1 siRNA; the expression of miR-503 was determined using real-time PCR. **d** NPCs were transfected with miR-NC or miR-503 mimics; the expression of MALAT1 was determined using real-time PCR. **e** The expression levels of miR-503 in IDD tissues and normal ones were determined by RT-qPCR. **f** The correlation between MALAT1 and miR-503 was analyzed using Spearman’s rank correlation analysis. The data are presented as mean ± SD of three independent experiments. *P < 0.05

In general, lncRNAs could interact with miRNAs, and affect miRNA-binding target genes and downstream signaling pathways. Therefore, we investigated whether miRNAs are involved in the effect of MALAT1 on IDD. Bioinformatic analysis showed that MALAT1 binds to miR-503 (Fig. 5a). Luciferase reporter experiments showed that the transfection of miR-503 mimics significantly inhibited the luciferase activity of wild-type MALAT1, but not mutated MALAT1 (Fig. 5b). Knockdown of MALAT1 significantly increased the level of miR-503 expression in NPCs (Fig. 5c), but the transfection of miR-503 mimics did not change the MALAT1 levels in NPCs (Fig. 5d). Additionally, the expression levels of miR-503 were detected in the clinical sample. As Fig. 5e shows, the miR-503 expression level was markedly higher in IDD tissue than in normal tissue. Furthermore, Spearman’s rank correlation analysis revealed that MALAT1 expression was negatively correlated with miR-503 (Fig. 5f). These data indicated that MALAT1 acts as a sponge for miR-503.

### MALAT1 inhibits miR-503 to regulate the function of NPCs and the MAPK pathway

**Fig. 6:**
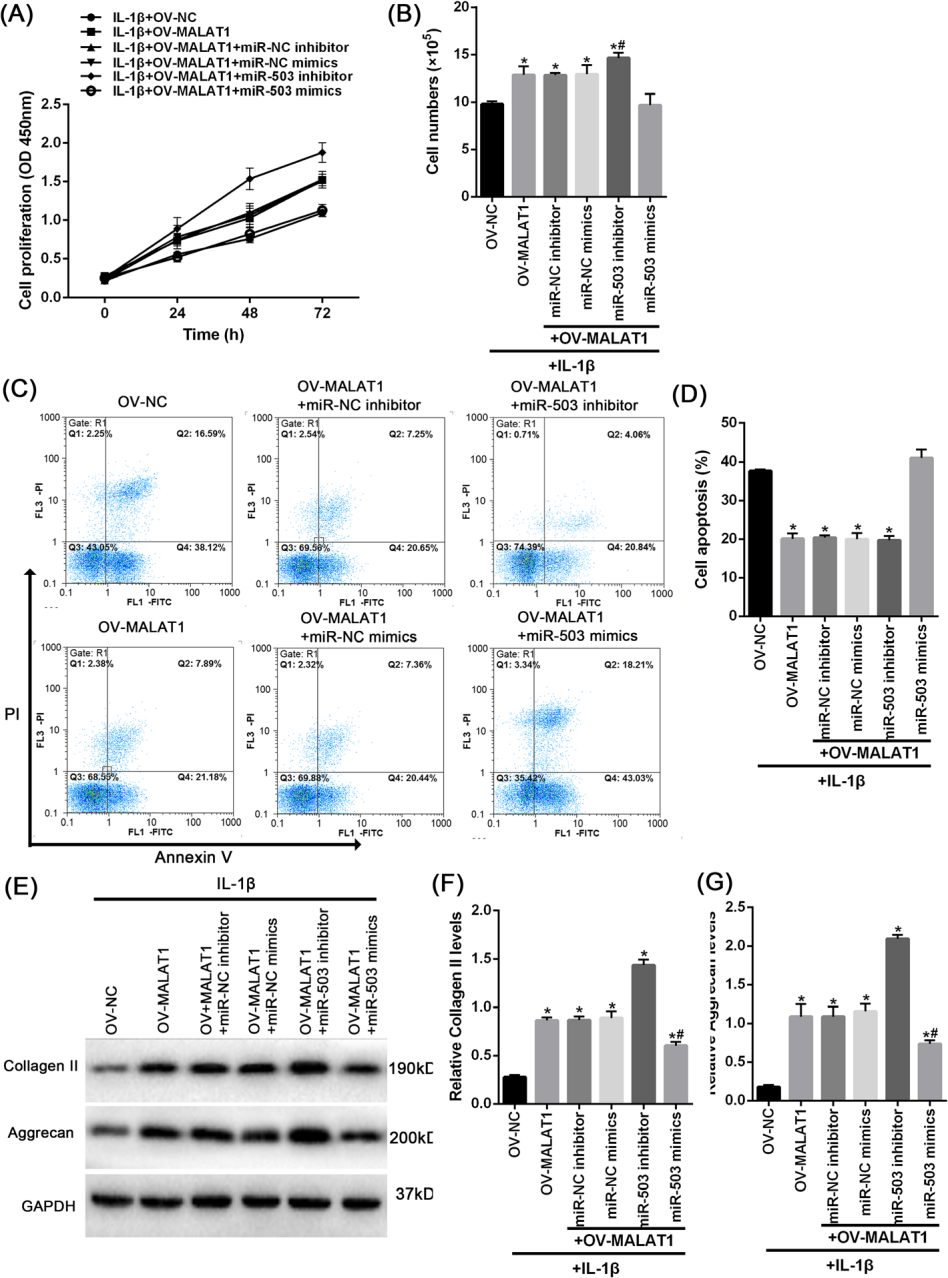
the effect of miR-503 on MALAT1 modulate the function of NPC NPCs were co-transfected with miR-503 mimics or inhibitor and OV-MALAT1 under IL-1β stimulation; (**a**) and (**b**) The cell viability was determined using CCK8 assays and cells counting. **c**-**d** The cell apoptosis was determined using Annexin V/PI stain. **e**-**g** The protein levels of Collagen II and Aggrecan were determined using Immunoblotting assays. **d** The protein levels of the MAPK pathway were determined using Immunoblotting assays. The data are presented as mean ± SD of three independent experiments. *P < 0.05, compared to control group; ##*P* < 0.01, compared to IL-1β + si-circSEMA4B group

We further verified the combined effect of MALAT1 and miR-503 on NPC proliferation, apoptosis, and ECM degradation, as well as the involvement of the MAPK pathway. Upon IL-1β stimulation, MALAT1 overexpression remarkably enhanced the NPC proliferation and NPC number (Fig. 6a-b) and reduced NPC apoptosis (Fig. 6c-d), but was attenuated by miR-503 mimics or aggravated by miR-503 inhibitor treatment. Furthermore, MALAT1 overexpression increased the protein levels of Collagen II and Aggrecan; the effect of MALAT1 overexpression was partially eliminated by miR-503 mimics. In contrast, miR-503 inhibitor increased Collagen II and Aggrecan protein levels (Fig. 6e-g).

**Fig. 7:**
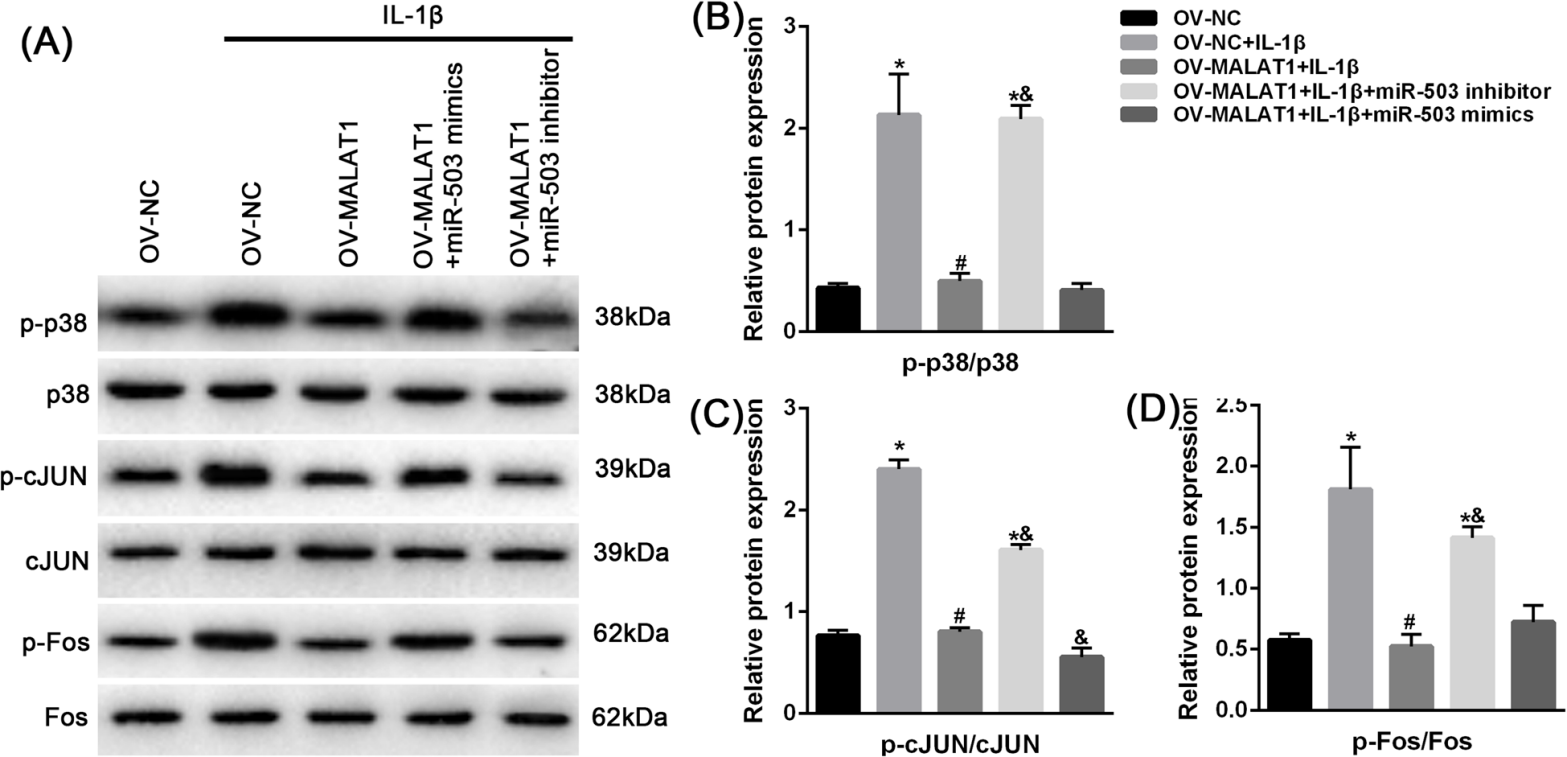
the effect of miR-503 on MALAT1 regulated MAPK signaling pathway **a** NPCs were transfected with a vector, upon IL-1β stimulation; the phosphorylated protein levels of p38, JUN, Fos were determined using Immunoblotting assays. The data are presented as mean ± SD of three independent experiments. *P < 0.05, compared to control group; #*P* < 0.05, compared to IL-1β group; & *P* < 0.05, compared to IL-1β + OV-MALAT1 group

Previous studies have shown that MALAT1 can affect the MAPK/AP1 signaling pathway [19], which is an important pathway for IVD [15]. Therefore, we examined the relationship between MALAT1, miR-503 and the MAPK pathway. Immunoblot results showed that overexpression of MALAT1 significantly reduced IL-1β-induced phosphorylation levels of p-fos, p-p38, and p-cJuN, and the effect of MALAT1 overexpression was partially eliminated by miR-503 mimics or aggravated by a miR-503 inhibitor (Fig. 7).

## Discussion

An increasing number of study have indicated the important roles of lncRNAs in IDD. In this study, we demonstrated that MALAT1 was downregulated in IDD tissues. MALAT1 overexpression promoted NPC proliferation and suppressed IL-1β-induced apoptosis, as well as IL-1β-induced degradation of ECM, indicating the protective effect of MALAT1 in IDD. Furthermore, we found that MALAT1 could sponge miR-503 and regulate the downstream factors of MAPAK pathway.

During the progression of IDD, NPCs produce excessive inflammatory mediators including TNF-α and IL-1β [20, 21], which is considered an important cause of IDD [22–24]. This phenomenon is observed with the senescence or apoptosis of NPCs, as well as the degradation of many protein components of the extracellular matrix (ECM) including Collagen II and Aggrecan. Therefore, we first focused on the expression of MALAT1 and ECM-degrading Collagen II/Aggrecan between normal and degenerative IVD tissues. The results showed that MALAT1 levels were decreased in IDD tissues, and Collagen II/Aggrecan were decreased. Moreover, in severe degeneration specimens, MALAT1, Collagen II, and Aggrecan levels were more downregulated. In Zhang et al. ‘s study [25], the expression level of MALAT1 was significantly reduced in NP cells isolated from IDD patients compared with controls. These findings suggested that reduced MALAT1 expression might participate in IDD development.

It is well known that NPCs are involved in resisting mechanical loads, and the synthesis of ECM is important to maintain spinal stability. The loss of NPCs correlates with the pathological process of IDD [26]. Therefore, we next focused on the effect of MALAT1 overexpression on NPC apoptosis and ECM degradation. Functionally, restored expression of MALAT1 partially attenuated the IL-1β-induced suppression of NPC proliferation, cell apoptosis, and degradation of ECM. At seven weeks after injection with a MALAT1-overexpressing lentiviral vector, rats appeared to have reduced spine curvature. Although no improved effect on the characterization of lumbar IDD was observed from X-ray, MALAT1 overexpression treatment improved the overall histological score shown by HE staining, which coincided with the degree of disc degeneration by MRI imaging. Under unbalanced dynamic and static forces of the spine, apoptosis in disc cells could be induced in the rat model [27]. In vitro, MALAT1 overexpression reduced IL-1β-induced apoptosis of NPCs. Additionally, we found that MALAT1 overexpression treatment could reduce TUNEL-positive NPCs in IDD rats. These findings indicate that suppressing NPC apoptosis by MALAT1 overexpression may contribute to the attenuation of IDD.

As mentioned, the primary mechanism of lncRNAs may serves as ceRNA by sponging miRNAs to regulate the downstream target mRNAs or related signal pathways. Based on a previous study and bioinformatic soft prediction, miR-503 might be a potential target of MALAT1 [12]. The luciferase reporter assay confirmed this prediction. Furthermore, miR-503 suppression mitigated the positive effects of MALAT1 on NPC cell proliferation and apoptosis induced by IL-1β, while miR-503 mimics enhanced this effect. A previous study showed that miR-503 plays an important role in the pathogenesis of postmenopausal osteoporosis [28]. In IVD, both Ji et al. [29] and Li et al. [30]‘s study demonstrated that miR-503 levels are significantly upregulated in IVD patients compared with controls, which is consistent with our results in this study.

Degeneration of the IVD appears to be mediated by several pathways. The MAPK pathway has been regarded as a crucial regulators of IVVD [31]. The MAPKs pathway can activate the downstream transcription factor AP1 (JUN, ATF, c-Fos, Maf) and then control many cellular processes including cell growth, differentiation, and apoptosis [32–34]. Several studes have shown that inhibition of MAPK/AP1 pathway is a potential therapeutic target for IDD. Our data showed that MALAT1 overexpression decreased IL-1β-upregulated p38, JUN and Fos levels, and miR-503 mimics or inhibitors attenuated or enhanced their phosphorylation level. These results are also consistent with previous studies [35–37], and imply that the mechanism governing MALAT1 may be the MAPK/AP1 signaling pathway. In addition, we found that miR-503 can antagonize the regulation of MAPK by MALAT1. Unfortunately, the mechanism governing the activity of miR-503 in the MAPK/AP1 pathway was not investigated. According to the existing evidence, miR-503 may not directly target genes in the MAPK/AP1 pathway, but indirectly regulate the MAPK/AP1 pathway by targeting such proteins as RANK and NFκB [28, 38]. We also predicted the targets of miR-503 through miRwalk 3.0 software [39] and found a few putative targets in given pathways, including MAPK, Wnt, Insulin pathway et al. (Table S1). This finding supports the potential role of MAPK/AP1 pathways in IVD degeneration.

## Conclusions

MALAT1 acts as a sponge and ceRNA for miR-503 and alleviates IL-1β-induced NPC apoptosis and degenerative processes through the MAPK signaling pathway. Our present study may help to elucidate the molecular mechanisms underlying IDD and provides a potentially effective therapeutic strategy for IDD.

## Methods

### Human tissue samples

This study was approved ethically by the First Affiliated Hospital of Kunming Medical University, and written informed consent was obtained from every participant. Degenerative NP tissues were obtained from 37 patients (average age 52.9 ± 9.6) with IDD, and normal NP tissues from 10 lumbar trauma patients (average age 21.7 ± 3.2) were excised by surgical resection, which was served as a control. The degree of disc degeneration was evaluated via a magnetic resonance imaging (MRI) scan according to the Pfirrmann grading classification. All specimens were collected within 3 h after disc excision, divided into two parts and frozen in liquid nitrogen for storage. All tissue samples were collected with written informed consent in accordance with the Declaration of Helsinki and with the approval of the Medical Ethics Committee of the First Affiliated Hospital of Kunming Medical University (Approval number: 2018061811, Date: 2018/06/18, Kunming, China). All of the animal experiments in this study were performed in accordance with the National Institutes of Health Guide for Care and Use of Laboratory Animals, and were approved by the laboratory animal ethical committee of Kunming Medical University.

### NPC isolation and culture

Normal human nucleus pulposus (HNP) tissues were gently separated from lumbar trauma patients under aseptic conditions, washed with D-Hank’s solution 3–5 times, and then cut into small pieces with ophthalmic scissors(< 1 mm^3^). Subsequently, tissues were placed overnight in 5 ml of 0.1% type II collagenase (GIBCO, NY, USA) at 37 °C for 8 h. The digested fluid was filtered through 200 meshes filters, followed by filtration and centrifugation at 1000 rpm for 5 min. The supernatant was removed, and the precipitate was suspended in 3 ml of medium and centrifuged at 1000 rpm for 5 min. The supernatant was removed again, and the NPCs were seeded into a culture flask in DMEM/F12 medium (GIBCO, NY, USA) containing 15% fetal bovine serum (FBS, GIBCO, NY, USA), 100 μg/ml streptomycin and 100 U/ml penicillin under 5% CO_2_ and saturated humidity at 37 °C. The culture medium was changed three times a week, and NPCs were subcultured at a ratio of 1:3 after reaching 80% confluence. Cell morphology was observed under an inverted microscope (DM6000B; Leica Microsystems, Japan). The concentration of HNPCs were adjusted to 1 × 10^4^/ml, and the cells were seeded into 24-well plates with glass coverslips for 48 h. Cells were fixed in formaldehyde for 20 min, followed by three washes with phosphate-buffered saline (PBS).

### Plasmid construction, dual-luciferase assays, and cell transfections

The lncRNA MALAT1 overexpression plasmid (OV-MALAT1) and negative control (OV-NC) were purchased from GenePharma (Shanghai, China). The following sequence of siRNA oligonucleotides (si-MALAT1) was used to knockdown MALAT1 expression: 5′-CACAGGGAAAGCGAGUGGUUGGUA-3′. The sequence of the noncoding control siRNA (si-NC) was 5′-UUCUCCGAACGUGUCACGU-3′. si-MALAT1, si-NC, mir-503 mimics, miR-503 inhibitor, and corresponding negative controls were purchased from RiboBio Co. (Guangzhou, China). Lipofectamine 2000 (Thermo Fisher Scientific, Waltham, USA) was used for transfection. Twenty-four hours before transfection, NP cells were seeded at 2 × 10^4^ cells/well in a 96-well plate. Cells were transfected with OV-NC or OV-MALAT1 using Lipofectamine 2000. Twenty-four hours after transfection, cells were treated with 150 ng/mL IL-1β for 6 h. Forty-eight hours later, NPCs were used for the following experiments.

### Luciferase assay

To verify whether there is a director interaction between MALAT1 and miR-503, a pmirGLO Dual-Luciferase miRNA target expression vector was used for 3′-untranslated region (UTR) luciferase assays (Promega, Madison, WI). 293 T cells were plated (5 × 10^4^ cells per well) in 24-well plates and cells in each well were cotransfected with miR-503 and wild type or mutant target sequences using Lipofectamine 2000. Cells were then harvested 48 h after transfection, and the activities of firefly and Renilla luciferases were measured by using the Dual-Luciferase Reporter Assay System with the miR-NC set at 1.0. In addition, cells were seeded in 6-well plates (1 × 10^6^ cells/well, 2 ml medium per well) and cultured normally for 24 h. Firefly luciferase activity was normalized to Renilla luciferase activity for each sample.

### RNA extraction and quantitative real-time PCR (qRT-PCR)

TRIzol reagent (Life Technologies, Gaithersburg, MD, USA) was used to extract total RNA from cells according to the manufacturer’s instruction. Then, cDNA was synthesized using 1 μg of total RNA as template and a RevertAidTM First Strand cDNA Synthesis Kit (Fermentas, Maryland, USA). Quantitative real-time PCR (qRT-PCR) analyses were performed with SYBR® Premix Ex Taq™(Takara, Japan) using a StepOne-Plus Real-Time PCR System (Applied Biosystems, USA). The PCR amplification included an initial denaturation at 95 °C for 1 min, 35 cycles of denaturation at 95 °C for 1 min, annealing at 60 °C for 2 min, and extension for 30 s, at 72 °C. Results of the log-linear phase of the growth curve were analyzed, and relative quantification was performed using the 2^-ΔΔCT^ method with GAPDH serving as a housekeeping gene. U6 snRNA was used as an internal control to normalize the expression levels of miRNAs. The PCR primers used were as follows: MALAT1, 5′- GACGGAGGTTGAGATGAAGC-3′ and 5′- ATTCGGGGCTCTGTAGTCCT-3′; IL-1β, 5′- GACGGAGGTTGAGATGAAGC-3′ and 5′- ATTCGGGGCTCTGTAGTCCT-3′; Collagen II, 5′- GACGGAGGTTGAGATGAAGC-3′ and 5′- ATTCGGGGCTCTGTAGTCCT-3′; Aggrecan, 5′- GACGGAGGTTGAGATGAAGC-3′ and 5′- ATTCGGGGCTCTGTAGTCCT-3′; GAPDH, 5′-GAAGGTGAAGGTCGGAGTC-3′ and 5′-GAAGATGGTGATGGGATTTC-3′.

### NPC proliferation by CCK-8

The viability of HNPCs was evaluated using Cell Counting Kit-8 (Dojindo, Kumamoto, Japan), according to the manufacturer’s instructions. In brief, cells were seeded in 96-well plates, and cultured for 8, 16, 24 h and stimulated with 150 ng/mL IL-1β (GIBCO, NY, USA) for 8, 16, and 24 h, respectively. NPCs were incubated with CCK-8 reagent for 1 h, and absorbance at 450 nm was measured with a microplate reader (Tecan, Männedorf, Switzerland).

### NPC apoptosis by Annexin V/propidium iodide (PI) staining

Cell apoptosis was detected using the Annexin V-FITC/PI staining method. After the cells were cultured for 24 h and stimulated with different concentrations of IL-1β (150 ng/mL) for the same culture time, they were washed twice with precooled PBS and digested with 0.25% trypsin, and the density was adjusted with DMEM to 1 × 10^6^ cells/mL. After fixing with precooled 70% alcohol for 24 h, the cells were centrifuged at 1000 r/min for 5 min, followed by two washes with PBS. Next, 100 μL of 1 × 10^6^ cells/mL cell suspension was added to 5 μL of Annexin V-FITC (Dojindo, Kumamoto, Japan) and 10 μL of PI, and the culture was incubated at room temperature for 15 min and washed twice with PBS. Cell apoptosis was analyzed using FACS Calibur Flow Cytometer (BD, USA). Each group had three replicates, and each process was repeated three times.

### Immunofluorescent staining

NPCs were plated on coverslips in 6-well plates at 2 × 10^5^ cells/well for 48 h. After cyclic stretching, the cells were harvested for fluorescence labeling of Collagen II. Briefly, cells were washed twice with PBS and fixed with 4% paraformaldehyde for 15 min. Then, after incubation with PBS containing 0.2% Triton X-100 for 10 min, cells were treated with PBS containing 5% bovine serum albumin (BSA) for 20 min to block nonspecific protein binding. After blocking, the cells were incubated overnight at 4 °C with primary antibodies (anti-Collagen II, 1:100, Santa Cruz) and then incubated with fluorescent secondary antibodies (1:200, Santa Cruz) at room temperature for 2 h. The staining results were visualized under a fluorescence microscope in the same field.

### Protein extraction and wWestern blot analysis

Total protein was extracted, and the protein concentration was quantified using a BCA protein assay kit (Beyotime Biotechnology, Jiangsu, China). A total of 20 μg of protein from each sample was used for Western blotting. The samples were separated by SDS-PAGE(10%) at 200 V for 50 min. After transferring the proteins onto polyvinylidene fluoride (PVDF) membranes, the blotting was performed at 300 mA for 45 min. After blocking with 5% (w/v) dry milk in TBS for 1 h at room temperature. Membranes were incubated with the primary antibodies. The primary antibodies used in this study included monoclonal anti-Collagen II (1:1000), Aggrecan (1:500) (Cell Signaling Technology, Beverly, MA), Fos (1:500), p38 (1:500), JNK (1:500) and their phosphorylated antibodies (1:500, Santa Cruz, CA, USA) and anti-GAPDH polyclonal antibody (1:2000 Santa Cruz, CA, USA) at 4 °C overnight. Then the membranes were incubated with HRP-conjugated anti-rabbit or anti-mouse antibody (1:10000, Cell Signaling Technology, Beverly, MA) for 2 h at room temperature. Finally, the blots were developed with an enhanced chemiluminescence kit (Millipore, Billerica, MA, USA), and the bands were quantified densitometrically using a Bio-Rad imaging system (Hercules, CA). GAPDH was used as the loading control.

### Establishment of the rat IVD model

Thirty-two male male Sprague–Dawley rats (350 g, aged three months) were obtained from Changsha Tian Qin Biotechnology Co., Ltd. (Changsha, Hunan, China). Rats were used for the experiments in vivo. Thirty-two rats were randomly divided into four groups: normal group (Control; *n* = 8), IVD model group without treatment (NT; n = 8), IVD model with the treatment of NC-overexpressing lentivirus (OV-NC; n = 8), and IVD model with the treatment of MALAT1- overexpressing lentivirus (OV-MALAT1; n = 8). All procedures were performed according to the National Institutes of Health Guide for Care and Use of Laboratory Animals and approved by the laboratory animal ethics committee of Kunming Medical University.

The rat model of IVD was built by annulus fibrosus (AF) needle puncture. In brief, general anesthesia was administered using 3.6% chloral hydrate and 10 mL/kg intraperitoneal. After successful anesthesia, SD rats were placed supine on the operation table, the limbs were fixed, and the tail was disinfected. According to the tail body surface markers of the rat, the tail bones C5 and C6 were determined. The middle part of the vertebral body was tilted by 45° to the left and right, and two diameters of 0.8 mm Kirschner wire were drilled. The carbon fiber loops were successively fixed on the crossed Kirschner wires, and the carbon fiber rods and springs were inserted. The experimental group was given a spring nut to fix the pressure, and the four springs were compressed to 10 mm/kg body weight (simulating the weight of the lumbar spine when the rat was walking upright). The compression length of the spring was controlled by a Vernier caliper; the control group did not pressurize the spring. After the operation, the two groups of animals were kept in a single cage, and penicillin 200,000 U was intramuscularly injected for three days to prevent infection.

SD rats were anesthetized with isoflurane gas (under a small animal anesthesia instrument). After anesthesia, the rats were placed on the operating table to fix the limbs. Under the guidance of fluoroscopy, in the three positions C6-C7, C8-C9, C10-C11, a 20 gauge needle was used to puncture the dorsal side, the puncture needle passed through the center of the intervertebral disc, until the opposite side, rotated 180°, and held for 10 s. The wound was wrapped with gauze after surgery and anti-infective treatment was carried out. One week after surgery, the rats were anesthetized again, and a small incision was made to the left of the puncture site to expose the position of the previous puncture. The intervertebral disc was pierced with a 33 gauge needle, followed by injection of OV-MALAT1 lentivirus or OV-NC lentivirus, and injections were repeated four weeks later.

### X-ray and MRI examination

At four weeks after injection, X-ray and MRI examinations were performed on all rats in the study. After examinations, five rats in each group were sacrificed by intraperitoneal injection of sodium pentobarbital. The tissues at the corresponding sites were harvested, fixed in 4% paraformaldehyde, and decalcified with 10% EDTA solution. The treated tissue was embedded in paraffin and sectioned in the sagittal plane. The disc was collected and stored at − 80 °C for further expression analysis.

### Histomorphology and TUNEL assay of the lumbar spine

After the MRI examination, the rats were killed by intraperitoneal administration of overdose pentobarbital sodium. Lumbar spines including L4–L6 levels were harvested en bloc and then fixed in 4% paraformaldehyde for 48 h, decalcified at 4 °C in 20% ethylenediamine tetraacetic acid for 5–7 weeks, embedded in paraffin, and sectioned (4 μm) along the midsagittal plane. Sections were used for hematoxylin-eosin (HE) or terminal deoxynucleotidyl transferase (TdT)-mediated dUTP nick end labeling (TUNEL). IDD was scored based on histomorphological features of HE-stained sections according to the classification system [28]. The average scores of L4–L5 and L5–L6 were recorded as the grade of lumbar IDD in each rat. Total and TUNEL-positive disc cells were counted below three to five noncontinuous high-power fields (magnification, × 400) in each of the two regions (outer or inner AF) from each of two discs per specimen and summed up. The percentage of TUNEL- positive disc cells compared with total disc cells was then calculated.

### Statistical analysis

Statistical differences between two groups were analyzed using Student’s t-test. Differences among three or more groups were analyzed using one-way analysis of variance and Tukey’s post hottest test. Data analysis was performed with GraphPad Prism 5 (Graphpad Software, La Jolla, CA, USA) and presented as the means ± SD. The difference was considered to be significant when *p* < 0.05.

## Supplementary information

**Additional file 1: Table S1** Significant pathways on putative target genes (3-UTR region) of miR-503.

## Data Availability

The datasets supporting the conclusions of this article are included within the article.

## Abbreviations

IDD: Intervertebral disc degeneration
NPCs: Nucleus pulposus cells
lncRNAs: Long non coding RNAs
ceRNA: Competing endogenous RNA
MALAT1: Metastasis-associated lung adenocarcinoma transcript-1
miRs: microRNAs
MRI: magnetic resonance imaging
HNP: Human nucleus pulposus
DMEM: Dulbecco’s modified Eagle’s medium
FBS: Fetal bovine serum
PI: Propidium iodide
IL-1β: Interleukin 1 beta
UTR: Untranslated region
SDS-PAGE: Sodium dodecyl sulfate polyacrylamide gel electrophoresis
PVDF: Polyvinylidene fluoride membranes
TUNEL: Terminal deoxynucleotidyl transferase (TdT)-mediated dUTP nick end labeling
