# Supplementary material for: LncRNA MALAT1 exhibits positive effects on nucleus pulposus cell biology in vivo and in vitro by sponging miR-503
Source: BMC Mol Cell Biol. 2020 Mar 30;21:23. doi: 10.1186/s12860-020-00265-2 (PMC7106590; doi:10.1186/s12860-020-00265-2)
Supplement: Supplementary file 1 — Additional file 1: Table S1 Significant pathways on putative target genes (3-UTR region) of miR-503. [file 12860_2020_265_MOESM1_ESM.docx]

Table S1 Significant pathways on putative target genes (3-UTR region) of miR-503

| Pathway Name | PathFg | PathBg | Fisher | BH |
| --- | --- | --- | --- | --- |
| Pathways in cancer | 215 | 330 | 6.86E-15 | 1.33E-12 |
| Axon guidance | 92 | 129 | 3.23E-10 | 6.21E-08 |
| MAPK signaling pathway | 170 | 272 | 6.21E-10 | 1.19E-07 |
| Insulin signaling pathway | 93 | 139 | 4.50E-08 | 8.55E-06 |
| Wnt signaling pathway | 100 | 152 | 5.26E-08 | 9.99E-06 |
| Long term potentiation | 53 | 71 | 1.62E-07 | 3.04E-05 |
| Chronic myeloid leukemia | 55 | 75 | 2.60E-07 | 4.88E-05 |
| Endocytosis | 116 | 187 | 5.50E-07 | 1.03E-04 |
| Melanogenesis | 69 | 102 | 1.29E-06 | 2.36E-04 |
| GnRH signaling pathway | 70 | 105 | 2.47E-06 | 4.49E-04 |
| Acute myeloid leukemia | 43 | 58 | 3.26E-06 | 5.93E-04 |
| Thyroid cancer | 25 | 29 | 3.34E-06 | 6.04E-04 |
| Glioma | 47 | 65 | 3.72E-06 | 6.74E-04 |
| Pancreatic cancer | 52 | 75 | 8.62E-06 | 1.53E-03 |
| Colorectal cancer | 58 | 86 | 1.03E-05 | 1.81E-03 |
| Renal cell carcinoma | 49 | 71 | 1.90E-05 | 3.33E-03 |
| Prostate cancer | 59 | 89 | 1.94E-05 | 3.39E-03 |
| Endometrial cancer | 38 | 52 | 2.13E-05 | 3.71E-03 |
| B cell receptor signaling pathway | 51 | 75 | 2.45E-05 | 4.27E-03 |
| Non small cell lung cancer | 39 | 54 | 2.61E-05 | 4.54E-03 |
| Lysosome | 75 | 121 | 5.44E-05 | 9.14E-03 |
| Neurotrophin signaling pathway | 79 | 129 | 6.36E-05 | 1.06E-02 |
| T cell receptor signaling pathway | 68 | 110 | 1.34E-04 | 2.19E-02 |
| mTOR signaling pathway | 37 | 53 | 1.36E-04 | 2.23E-02 |
| Gap junction | 57 | 90 | 1.80E-04 | 2.92E-02 |
| p53 signaling pathway | 45 | 68 | 1.96E-04 | 3.15E-02 |
| ErbB signaling pathway | 56 | 89 | 2.63E-04 | 4.20E-02 |
| Adherens junction | 49 | 76 | 2.69E-04 | 4.31E-02 |
| Phosphatidylinositol signaling system | 49 | 76 | 2.69E-04 | 4.31E-02 |
| VEGF signaling pathway | 50 | 78 | 2.88E-04 | 4.58E-02 |

PathFg, stand for number of genes predicted as putative targets in a given pathway; PathBg, stand for number of genes in a given pathway; BH, depict the significant pathways after pvalue adjustment (multiple testing method or FDR correction).
